# Supplementary material for: Testing the knowledge of artificial intelligence chatbots in pharmacology: examples of two groups of drugs
Source: PeerJ Comput Sci. 2025 Jul 15;11:e2954. doi: 10.7717/peerj-cs.2954 (PMC12453646; doi:10.7717/peerj-cs.2954)
Supplement: Supplemental Information 1 [file peerj-cs-11-2954-s001.docx]

Question: 1. In invasive infection caused by Candida albicans administration of which drug/drugs may be recommended? Answers: a. voriconazole b. terbinafine c. caspofungin d. correct answers are a and c

Question: 2. In invasive aspergillosis administration of which drug may be recommended? Answers: a. fluconazole b. terbinafine c. natamycin d. caspofungin

Question: 3. Clinically significant drug interaction involving the P450 isoforms may occur in case of simultaneous administration of: Answers: a. terbinafine and amitriptyline b. fluconazole and carbamazepine c. amphotericin B and metformin d. correct answers are a and b

Question:4. Among undermentioned drugs the lowest bioavailability after oral administration has: Answers: a. isavuconazonium b. fluconazole c. itraconazole d. voriconazole

Question: 5. In urinary tract infection caused by Candida krusei administration of which drug may be recommended? Answers: a. voriconazole b. fluconazole c. amphotericin B d. micafungin

Question: 6. Conventional formulation of amphotericin B when compared to liposomal formulations has: Answers: a. lower nephrotoxicity b. higher hepatotoxicity c. better penetration to central nervous system d. significant effectiveness in treatment of urinary tract infections caused by susceptible strains

Question: 7. Echinocandins: Answers: a. may be responsible for clinically significant kidney damage b. are characterized by broad antifungal spectrum – similarly to amphotericin B c. are potent P450 isoforms inhibitors d. do not require dosage modifications in patients with renal insufficiency

Question: 8. In patients infected with HIV and Pneumocystis jirovecii administration of which drug may be recommended? Answers: a. cotrimoxazole b. voriconazole c. flucytosine d. micafungin

Question: 9. Choose correct sentence concerning itraconazole’s interaction: Answers: a. increases omeprazole’s bioavailability b. lowers eplerenone’s effectiveness c. increases fentanyl’s adverse effects d. shows synergistic effect with naproxen’s anti-inflammatory profile of action

Question: 10. Choose correct sentence about antifungal therapy: Answers: a. pharmacotherapy of onychomycosis should not last longer than 14 days b. invasive mycosis always requires systemic pharmacotherapy c. preventive antifungal therapy should always come along with prolonged antibacterial therapy d. dermatophytosis of the scalp is always indication to local drug application

Question: 11. Azole anitfungals’ mechanism of action is based on inhibition: Answers: a. DNA gyrase b. lanosterol 14α-demethylase c. 1,3-Beta-glucan synthase d. cytosine deaminase

Question: 12. Among azoles’ adverse effects one can mention increased risk of: Answers: a. hepatitis b. auricular fibrillation and cardiac arrest c. esophageal candidiasis d. osteoporosis

Question: 13. Choose correct sentence concerning amphotericin B: Answers: a. flucytosine weakens its antifungal properties b. is characterized by high bioavailability when administered orally (90%) c. when applied as liposomal formulation is not harmful for bone marrow d. is characterized as a drug with broad-spectrum antifungal activity

Question: 14. In Poland Amphotericin B is available in form of: Answers: a. tablets b. powder to prepare oral suspension c. concentration to prepare intravenous infusion d. solution for intramuscular injection

Question: 15. Choose antifungal drug which is applied only locally: Answers: a. ketoconazole b. terbinafine c. anidulafungin d. fluconazole

Question: 16. Amorolfine: Answers: a. inhibits the synthesis of glucan b. is characterized by narrow-spectrum antifungal activity limited to Candida spp. c. is available in form of a nail lacquer d. is drug of first choice in case of esophageal mycosis

Question: 17. Flucytosine: Answers: a. inhibits nucleic acids synthesis b. acts synergistically with amorolfine c. because of its antifungal activity spectrum is widely applied in life-threatening invasive mycoses d. is not absorbed from gastrointestinal tract

Question: 18. Ergosterol synthesis is inhibited by: Answers: a. terbinafine b. amorolfine c. posaconazole d. corrects answers are a, b, and c

Question: 19. Voriconazole: Answers: a. because of genetic polymorphisms concerning hepatic metabolism can manifest increased toxicity in some patients b. can be administered orally c. can cause visual disturbances d. corrects answers are a, b, and c

Question: 20. Choose correct combination drug and its adverse effect: Answers: a. voriconazole - long QT syndrome b. nystatin – hyperkalemia c. ciclopirox – liver function disorders d. flucytosine – hypoglycemia

Question: 21. Echinocandins’ mechanism of action depends on inhibition: Answers: a. DNA gyrase b. lanosterol 14α-demethylase c. 1,3-Beta-glucan synthase d. cytosine deaminase

Question: 22. During azoles administration patients are exposed to increased risk of: Answers: a. clinically significant drug interactions b. auricular fibrillation and cardiac arrest c. esophageal candidiasis d. osteoporosis

Question: 23. Choose correct sentence concerning amphotericin B: Answers: a. flucytosine weakens its antifungal properties b. is characterized by low bioavailability when administered orally c. when applied as liposomal formulation is not harmful for bone marrow d. is characterized as a drug with narrow-spectrum antifungal activity (Aspergillus spp., Candida spp.)

Question: 24. Choose antifungal drug which can be applied locally and in systemic therapy: Answers: a. ketoconazole b. terbinafine c. anidulafungin d. itraconazole

Question: 25. In invasive infection caused by Candida albicans administration of which drug/drugs may be beneficial? Answers: a. fluconazole b. terbinafine c. anidulafungin d. correct answers are a and c

Question: 26. In invasive aspergillosis administration of which drug may be recommended? Answers: a. voriconazole b. caspofungin c. natamycin d. correct answers are a and b

Question: 27. Clinically significant drug interaction involving the P450 isoforms may occur in case of simultaneous administration of: Answers: a. terbinafine and carbamazepine b. itraconazole and clarithromycin c. amphotericin B and metformin d. correct answers are a and b

Question: 28. Administration of which drug is recommended in urinary tract infection caused by Candida krusei? Answers: a. caspofungin b. fluconazole c. amphotericin B d. voriconazole

Question: 29. Echinocandins: Answers: a. may be responsible for clinically significant kidney damage b. are characterized by broad antifungal spectrum – similarly to amphotericin B c. cannot be characterized as a class of antifungal drugs that cause clinically significant interactions by affecting P450 isoforms d. require dosage modifications in patients with renal insufficiency

Question: 30. Choose correct combination drug and its adverse effect: Answers: a. voriconazole - long QT syndrome b. nystatin – skin pigmentation c. ciclopirox – kidney function disorders d. flucytosine – hypoglycemia
