# Supplementary material for: Testing the knowledge of artificial intelligence chatbots in pharmacology: examples of two groups of drugs
Source: PeerJ Comput Sci. 2025 Jul 15;11:e2954. doi: 10.7717/peerj-cs.2954 (PMC12453646; doi:10.7717/peerj-cs.2954)
Supplement: Supplemental Information 2 [file peerj-cs-11-2954-s002.docx]

Question: 1. Choose correct sentence concerning statins: Answers: a. their hypolipidemic activity is dependent on the dose b. they act as anti-inflammatory agents, so they reduce pain c. they have pleiotropic effects which means they lower blood pressure and slow down the heart rate d. they can be used only in monotherapy

Question: 2. Before therapy of statins begin, it is mandatory is to determine in the blood: Answers: a. transaminases activity b. TSH concentration c. potassium concentration d. calcium concentration

Question: 3. Significant risk of transaminases activity increase is possible in case of administration of: Answers: a. rosuvastatin b. evolocumab c. ciprofibrate d. correct answers are a and c

Question: 4. Intracellular mechanism of action is characteristic for: Answers: a. lovastatin b. inclisiran c. colesevelam d. correct answers are a and b

Question: 5. Choose correct combination drug-adverse effect: Answers: a. colesevelam – dysfunction of vitamin K absorption b. bempedoic acid – increased concentration of uric acid in blood (hyperuricemia) c. lomitapide - hepatic steatosis d. correct answers are a, b, and c

Question: 6. Decrease of serum LDL concentration can be achieved by: Answers: a. decreasing the absorption of cholesterol in small intestine b. decreasing cholesterol synthesis in hepatocytes c. increasing the density of LDL receptors in the surface of hepatocytes d. Correct answers are a, b, and c

Question: 7. Choose correct sentence concerning pleiotropic effects of statins: Anserws: a. they increase activity of nitric oxide synthase b. they decreas cholesterol synthesis in hepatocytes c. they increase the density of LDL receptors in the surface of hepatocytes d. Correct answers are a, b, and c

Question: 8. The strongest statin in means of decreasing LDL concentration (regarding equivalent dose) is: Answers: a. rosuvastatin b. fenofibrate c. lovastatin d. fluvastatin

Question: 9. During pregnancy you may administer: Answers: a. colesevelam b. fenofibrate c. lovastatin d. rosuvastatin

Question: 10. Contraindication for all statins is: Answers: a. pregnancy b. renal insufficiency c. hyperthyroidism d. cardiac arrhythmias

Question: 11. Statins’ mechanism of actions that leads to lowering low density lipoprotein (LDL) levels is an effect of inhibition: Answers: a. cholesterol absorption in the gastrointestinal track b. 3-hydroxy-3-methyl-glutaryl-coenzyme A reductase (HMG-CoA reductase) c. proprotein convertase subtilisin/kexin type 9 d. ATP-citrate lyase

Question: 12. The increased risk of rhabdomyolysis during statins therapy is present in case of: Answers: a. liver diseases b. alcohol abuse c. women in elderly age (above 65 years old) d. correct answers are a, b, and c

Question: 13. The increased risk of myopathy and rhabdomyolysis is present case of simultaneous administration of simvastatin and: Answers: a. itraconazole b. clarithromycin c. acetylsalicylic acid d. correct answers are a and b

Question: 14. Ezetimibe: Answers: a. can be administered simultaneously with statins b. is drug of first choice in prophylaxis of cardiovascular events c. inhibits absorption of cholesterol in large intestine d. does not affect liver transaminases activity

Question: 15. During therapy with the use of ezetimibe may occur: Answers: a. pancreatitis b. myopathy c. erythema multiforme d. Correct answers are a, b, and c

Question: 16. Evolocumab: Answers: a. is a drug that inhibits proprotein convertase subtilisin/kexin type 9 b. can be a cause of a flu-like syndrome c. is a biotechnological drug d. correct answers are a, b, and c

Question: 17. Choose correct sentence about fibrates: Answers: a. they can be safely administered together with statins b. they are specifically effective in lowering triglyceride levels c. they can cause phototoxic reactions d. correct answers are b and c

Question: 18. Choose correct combination drug-mechanism of action: Answers: a. ciprofibrate – affecting transcription factors that regulate lipids and lipoproteins metabolism b. colesevelam – increasing reabsorption of bile acids-cholesterol complex to circulation c. inclisiran – inhibition of LDL receptors’ degradation d. bempedoic acid – decreasing cholesterol synthesis in skeletal muscles

Question: 19. Omega-3 fatty acids: Answers: a. have proved by clinical trials, significant, beneficial effect on cardiovascular disease risk b. increase the risk of bleeding in patients treated with acetylsalicylic acid c. their mechanism of action is based on inhibition of both HMG-CoA reductase and ATP-citrate lyase d. decrease the risk of pancreatic cancer

Question: 20. Myopathy can be caused by: Answers: a. atorvastatin b. ciprofibrate c. ezetimibe d. correct answers are a, b, and c

Questions: 21. Statins’ mechanism of actions that leads to lowering low density lipoprotein (LDL) levels is an effect of inhibition: Answers: a. cholesterol absorption in the gastrointestinal track b. proprotein convertase subtilisin/kexin type 9 c. 3-hydroxy-3-methyl-glutaryl-coenzyme A reductase (HMG-CoA reductase) d. liver transaminases activity

Questions: 22. Choose correct sentence about statins: Answers: a. their hypolipidemic activity is not dependent on the dose b. they act as anti-inflammatory agents, so they reduce pain c. they have pleiotropic effects which means they lower blood pressure and slow down the heart rate d. they can be used in monotherapy as well as in combination with other hypolipidemic drugs

Question: 23. The increased risk of rhabdomyolysis during statins therapy is present in case of: Answers: a. renal insufficiency b. life-threatening bacterial infection c. alcohol abuse d. correct answers are a, b, and c

Question: 24. The increased risk of myopathy and rhabdomyolysis is present case of simultaneous administration of simvastatin and: Answers: a. posaconazole b. amiodarone c. acetylsalicylic acid d. correct answers are a and b

Question: 25. Ezetimibe: Answers: a. cannot be administered simultaneously with statins b. is drug of first choice in prophylaxis of cardiovascular events c. inhibits absorption of cholesterol in small intestine d. does not affect liver transaminases activity

Question: 26. Evolocumab: Answers: a. is a drug that inhibits proprotein convertase subtilisin/kexin type 9 b. can cause rhabdomyolysis c. is a biotechnological drug d. correct answers are a and c

Question: 27. Which drug can be used during pregnancy? Answers: a. colesevelam b. ciprofibrate c. ezetimibe d. simvastatin

Question: 28. Choose correct combination drug-mechanism of action: Answers: a. fenofibrate – affecting transcription factor that regulates lipids and lipoproteins metabolism b. colesevelam – inhibition of bile acids reabsorption in the intestines c. inclisiran – inhibition of LDL receptors’ degradation d. correct answers are a and b

Questions: 29. Omega-3 fatty acid: Answers: a. have proved by clinical trials, significant, beneficial effect on cardiovascular disease risk b. decrease the risk of bleeding in patients treated with acetylsalicylic acid c. their mechanism of action is poorly understood d. decrease the risk of pancreatic cancer

Question: 30. Decrease of serum LDL concentration can be achieved by: Answers: a. increasing the absorption of cholesterol in small intestine b. decreasing cholesterol synthesis in hepatocytes c. increasing the density of LDL receptors in the surface of hepatocytes d. Correct answers are b and c
